# Injuries and concussions among young children, ages 5-11, playing sports in recreational leagues in Florida

**DOI:** 10.1371/journal.pone.0216217

**Published:** 2019-05-15

**Authors:** Karen D. Liller, Barbara Morris, Yingwei Yang, Omonigho M. Bubu, Brad Perich, Jessica Fillion

**Affiliations:** 1 University of South Florida, Tampa, Florida, United States of America; 2 Florida Hospital, Wesley Chapel, Florida, United States of America; 3 Wheaton College, Wheaton, Illinois, United States of America; Instituto Politecnico de Viana do Castelo, PORTUGAL

## Abstract

**Background:**

The specific research aims of this study included: 1) Conduct an epidemiologic analysis of recreational sports injuries among 1500 children, ages 5–11 in Florida: and 2) Utilize the computerized pediatric concussion tool from ImPACT Applications, Inc. for baseline and follow-up testing to better understand these injuries. This research followed a prospective surveillance design utilizing a large cohort of children, ages, 5–11, who play recreational football, soccer, and baseball/softball in Florida. The study venue was a large athletic facility in Hillsborough County, Florida. The sports observed were soccer (girls’ and boys’), baseball, softball, and football. Internal and external advisory boards were consulted throughout the study.

**Methods:**

Certified Athletic Trainers (ATCs) were hired to use High School Reporting Information Online (RIO) for injuries and the Ipad-administered pediatric concussion tool developed by ImPACT Applications, Inc for baseline/follow-up concussion data.

**Results:**

Over the course of the project, 26 RIO-reported injuries were reported. Football and soccer produced the greatest rate of injuries. There were 12 concussions which comprised nearly half of all the RIO injuries (46%). We conducted 882 baseline concussion tests and 13 follow-up tests over the 2 years.

**Conclusions:**

To the best of our knowledge, this is the first time data have been collected and reported on sports injuries in the study population. Future studies built on these findings will allow for the development of targeted guidelines and interventions for coaches, players, and parents so sports injury-related morbidity and mortality decrease in our youngest athletes.

## Introduction

Injuries related to sports and recreation are a substantial public health problem [1]. While it is important to promote physical activity in children and adolescents, sports injuries are also important to assess. [2]. Based on the National Health Interview Survey findings for individuals 5 years of age and older from years-2011-2014, sports and recreational injury rates were higher among males, children aged 5–14 years, and non-Hispanic white persons than for their counterparts [2]. General exercise was the most frequently mentioned activity associated with sports- and recreation-related injuries, but types of activities varied across sex and age groups. Body regions injured while engaging in sports and recreation activities included the lower extremity (42.0%), upper extremity (30.3%), and head and neck (16.4%) [2].

Data from the Centers for Disease Control and Prevention show that recreational activities, including sports, account for about 3.2 million visits to emergency rooms each year for children aged 5–14 years [3]. Also, injuries from organized and unorganized sports produce 775,000 emergency department visits annually for children in this same age group. In fact, sports-related injuries are the leading cause of emergency department visits in 12-17-year-olds [3].

Another study showed that sports related injuries in US EDs varied per sport. Leading sports for injuries were football, basketball, soccer and baseball, occurring among children aged 5–18 years during 2001–2013 [4]. In terms of head injuries in children, results of the 2016 National Health Interview Survey showed that parents reported about 7% of children, age 3–17 have ever had a significant head injury. Being male, non-Hispanic white, and at an increasing age were associated with parents reporting more significant head injuries among their children. The report showed that about 1 in 10 children age 15–17 ever had a significant head injury. The majority of children who ever had a significant head injury only had one such injury (81.3%) and only 18.7% had two or more [5]. A new CDC Pediatric Mild Traumatic Brain Injury (mTBI) Guideline has been published that addresses 19 sets of clinical recommendations related to diagnosis, prognosis, management, and treatment of mTBIs [6]. Results of a study that used electronic health records data from the Children’s Hospital of Philadelphia showed that from 2012–2014, 67% of concussions reported in children ages 5–11 were sports-related [7]. This percentage showed increases as children aged (77% for ages 12–14 and 73% for ages 15–17).

Although concussions among younger athletes is an important public health issue, much remains unknown on the long-term impact of sports-related concussion in children and how parents and health care providers can appropriately manage them [8, 9]. A recent study aiming to explore parents’ knowledge of concussion among young football players aged 5–12 years old found that misconceptions about concussion commonly existed among parents [10].

Moreover, healthcare providers may need reliable tools to assess head injuries and concussions. In a recent national study of health care national providers who saw pediatric patients in their practice, over half (59%) reported that they diagnosed or managed pediatric patients with a mTBI (or concussion) within the last 12 months, however only 44.4% reported feeling very prepared to make decisions pertaining to the patients’ safe return to school and sports after the mTBI [11]. Some researchers assert that clinical decision support tools, such as computerized neuropsychological tests with baseline and post-concussion assessments, would be useful for healthcare providers to identify symptoms and make management decisions with sports-related concussion [11–13].

To date, research on sports injuries has largely been focused on high school and collegiate athletes. For child athletes, many sports/recreational activities are not organized for reporting injuries, so almost no data for this group have been collected [14]. No effective prevention strategies can be properly developed without the knowledge of the mechanisms related to these injuries, including concussions. To address this need, we conducted a two-year epidemiologic analysis of recreational sports injuries among children ages 5–11 who play football, soccer, baseball and softball in Florida.

This study’s aims included the following: 1) Conduct an epidemiologic analysis of recreational sports injuries among 1500 children, ages 5–11 in Florida: and 2) Utilize the new computerized pediatric concussion tool from ImPACT Applications, Inc. for baseline and follow-up testing to better understand these injuries.

## Materials and methods

### Participants

This research followed a prospective surveillance design utilizing a large cohort of children, ages, 5–11, who play recreational football, soccer, and baseball/softball at a large athletic facility in Hillsborough County, Florida. Data were collected over a two-year period. For each year of the study over 1,500 athletes (1,511 in Year 1 and 1,543 in Year 2) participated in the sports provided.

Certified athletic trainers (ATCs) were hired to use High School Reporting Information Online (RIO) to capture athletic exposure (number of athlete practices and number of athlete competitions per week), injury (body site, diagnosis, severity, etc) and injury event (mechanism, activity, position/event, field/court location, etc) data [15]. Data were sent to the researchers from the RIO central office weekly. Due to some scheduling conflicts, more ATCs were available in Year 1 than in Year 2 for the collection of injury and concussion data. The University of South Florida Institutional Review Board approved the study. No identifiers were available to the researchers.

In order for an injury to be counted in the RIO database the following criteria must have been met: The injury: 1) occurred because of participation in competition and practice in recreational leagues; 2) required medical attention by a team physician, certified athletic trainer, personal physician, or emergency department/urgent care facility; 3) resulted in restriction of the athlete’s participation for one or more days beyond the day of injury; and 4) any fracture, concussion, dental, or heat injury regardless of whether it resulted in restriction of the athlete’s participation [15]. We also extensively utilized the Ipad-administered pediatric concussion tool developed by ImPACT Applications, Inc (test version of ImPACT Pediatric) for baseline/follow-up concussion data. ATCs were trained in the use of the ImPACT tool and working relationships were established with the athletic complex, athletic directors, and coaches before the start of the study. Advisory groups comprised of practitioners, researchers, and a neuropsychologist were available for review and discussion of findings.

Baseline neurocognitive data were collected with ImPACT Pediatric on available and consenting athletes with follow-up testing as requested by physicians and/or parents. Children were tested before participating in practices or games to avoid fatigue when responding [16].

### ImPACT pediatric

ImPACT Pediatric is the only FDA-cleared concussion assessment tool for ages 5–11. Test providers must complete the appropriate online training and have a basic level of knowledge regarding the medical assessment of concussion and neurocognitive factors [16]. Post-injury testing must be done by properly trained and licensed healthcare professionals with specific knowledge and experience in interpreting neurocognitive test results [16]. Administration time is about 15–20 minutes. The test is to be administered by professionals specifically trained, including psychologists, ATCs, and nurses. Reliability testing to date has shown that test-retest correlation scores are high with intra-class correlations ranging from .46-.83 for test components, including word lists, design rotation, stop & go, memory touch, and picture match. ImPACT Pediatric was standardized with a sample of 5-11-year-old children from 2012–2015 in several states, including Georgia, Maryland, Michigan, New York, New Jersey, Pennsylvania, Texas, and Virginia [16]. The ImPACT Pediatric manual shows the test has good validity, including face and concurrent validity [16].

### Data analysis

Data analysis included a series of descriptive and analytical statistical analyses,. For Years 1 and 2, injury rates, diagnoses, and mechanisms (shown as frequencies and percentages) were reviewed. Injury rates were defined as the ratio of unweighted case counts per 1000 athletic exposures.

The analytical analyses of ImPACT Pediatric results were conducted largely in Year 1 due a greater number of tests conducted during that period. For this analysis, an exploratory factor analysis with varimax rotation using Eigenvalues >1.0 for factor extraction was conducted with and showed a four-factor solution of word memory (word items), sequencing/attention (memory touch), visual memory (picture match average taps and average time), and reaction time (stop and go average time and design rotation number [16]. To examine the effect of age on the ImPACT scores, a linear regression model was utilized. The model was adjusted for sex and interaction terms for age and sex were examined. Statistical significance for all tests conducted was determined using Bonferroni correction (*p* ≤ .0125) since we conducted four separate independent t-tests and four separate linear regressions, one for each Pediatric ImPACT composite score. For the ImPACT scores, all analyses were conducted with SAS 9.4.

## Results

### YEAR 1

Table 1 shows the injuries and injury rates and exposures for Years 1 and 2 of the study. For Year 1 of the study, there were 1,511 participating athletes and 18 injuries occurred in practices and competitions. Athletes varied in ages from 8–10 for Year 1 and 9–11 in Year 2. Football had the highest rate of injuries in both competitions and practices (1.18 and .68, per 1000 athlete-exposures respectively) [15]. Table 1 shows that softball, girls’ soccer and boys’ soccer also had high rates in competitions [15] in Year 1. Some results of Year 1 have been previously published [15, 16].

**Table 1:** Year 1 and Year 2 sports-related injuries in competitions (C) and practices (P) among 5–11 year old athletes playing in recreational leagues in Hillsborough County, Florida (N = 26).

Sports	Year 1			Year 2		
	Athletic Exposures	Injuries N = 18	Injury Rate (per 1000)	Athletic Exposures	Injuries N = 8	Injury Rate (per 1000)
Football-C	847	1	1.18	638	0	0
Football-P	2923	2	0.68	2954	0	0
Softball-C	3192	2	0.63	2731	0	0
Softball-P	3004	1	0.33	3668	1	0.27
Soccer (Girls’)-C	2982	2	0.67	2594	3	1.16
Soccer (Girls’)-P	4730	1	0.21	5275	0	0
Soccer (Boys’)-C	5016	3	0.60	4421	1	0.23
Soccer (Boys)’-P	8058	2	0.25	10518	3	0.29
Baseball-C	9446	4	0.42	9122	0	0
Baseball-P	6995	0	0	5156	0	0

In Year 1, we conducted 663 ImPACT Pediatric baseline tests and five follow-ups [15]. Follow-up findings were very similar to baseline findings. Pertaining to Year 1 RIO injuries, most occurred in males (66.7%) and took place during competition (66.7%). The leading types of injuries were concussions (22.2%) and fractures (22.2%), followed by contusions (16.7%), and ligament sprains (16.7%) [15]. Boys’ soccer had 75% of the concussions, girls’ soccer had the majority of contusions, and baseball had 50% of the fractures. The head/face (22.2%) was the most frequently injured body part across sports, followed by the ankle, elbow, knee, shoulder, and thigh/upper leg (11.1% for each) [15]. Table 2 shows injury diagnosis by sport for Year 1.

**Table 2:** Year 1 Injury diagnoses by sport in RIO (N = 18).

Diagnosis	Boys’ Soccer		Baseball		Football		Girls’ Soccer		Softball		Total	
	N	%	N	%	N	%	N	%	N	%	N	%
Concussion	3	16.67	0	0.00	0	0.00	0	0.00	1	5.56	4	22.22
Fracture	1	5.56	2	11.11	1	5.56	0	0.00	0	0.00	4	22.22
Contusion	0	0.00	0	0.00	0	0.00	2	11.11	1	5.56	3	16.67
Ligament sprain	0	0.00	1	5.56	1	5.56	1	5.56	0	0.00	3	16.67
Dislocation	0	0.00	1	5.56	0	0.00	0	0.00	0	0.00	1	5.56
Heat illness/injury	0	0.00	0	0.00	0	0.00	0	0.00	1	5.56	1	5.56
Laceration	1	5.56	0	0.00	0	0.00	0	0.00	0	0.00	1	5.56
Separation	0	0.00	0	0.00	1	5.56	0	0.00	0	0.00	1	5.56
**Total**	5	27.78	4	22.22	3	16.67	3	16.67	3	16.67	18	100.00

The leading injury mechanisms were contact with another person (33.3%) and playing apparatus (33.3%), followed by playing surface (22.2%) [15]. Most injuries were new (88.9%). More than half of injured athletes returned to play within 21 days (55.6%), and none of them required surgery. All the injuries were initially assessed by the onsite ATC, and managed by general physicians-pediatricians (55.6%) and/or the ATC (22.2%).

Results from the independent t-tests for the baseline ImPACT Pediatric tests showed there was a significant difference in Visual Memory scores with sex (p = 0.01) with females performing better as shown by fewer number of taps and faster average time to respond [16]. The adjusted linear models showed significant effects of age on three of the neurocognitive domains. These included sequencing/attention, word memory, and visual memory [16]. No significant age effects were seen with reaction time. Overall, participants performed better with increasing age [16]. See Table 3 for the results of the linear regression for age and sex.

**Table 3:** Linear regression for age and sex (N = 657).

	Beta Estimate	Standard Error	t-value	p-value
**Sequencing/Attention**				
Age	0.46	0.04	12.57	<0.0001*
Sex (Ref: Male)				
Female	0.56	1.03	-0.54	0.59
Age* Sex	0.02	0.08	-0.27	0.79
**Word Memory**				
Age	0.2	0.02	11.24	<0.0001*
Sex (Ref: Male)				
Female	-0.81	0.5	-1.62	0.11
Age* Sex	0.02	0.04	0.55	0.58
**Visual Memory**				
Age	-1.54	0.16	-9.32	<0.0001*
Sex (Ref: Male)				
Female	-0.71	4.64	-0.15	0.88
Age* Sex	-0.46	0.35	-1.30	0.19
**Reaction Time**				
Age	0.03	0.02	1.49	0.14
Sex (Ref: Male)				
Female	-0.53	0.48	-1.10	0.27
Age* Sex	0.0005	0.04	0.15	0.88

*p < .0125

### YEAR 2

In Year 2 there were 1543 players and we conducted 219 ImPACT baseline tests with eight follow ups. Follow-up findings were very similar to baseline findings. There were eight RIO injuries that occurred in year 2, all concussions. See Table 1 for injury and exposure rates. All injuries involved the head and face, were new and none required surgery. Fifty percent of concussions took place during practices and 50% took place during competitions. Fifty percent of the concussions took place in boys’ soccer, followed by 37.5% in girls’ soccer, and 12.5% in softball. Fifty percent of the concussions occurred in males, and 50% occurred in females. The majority of injuries took place during the regular season (87.5%). Methods of evaluation were largely conducted by the athletic trainer (55.56%) and/or physicians (44.49%). Approximately 57% of the cases returned to activity within 10–21 days and for 43% the season ended before return to play could occur. The leading mechanism of injury was contact with a playing apparatus (N = 4; 50%) followed by contact with another person (N = 2; 25%) and contact with the playing surface (N = 2; 25%).

## Discussion

To date, our study produced some of the only injury and concussion findings on children, ages 5–11, playing sports in recreational leagues. The results of our project have shown that children, between the ages of 5–11, who play sports in recreational leagues do get injured and nearly half of these injuries are concussions. Over the course of the two years of this project 26 RIO-reportable injuries occurred, of which nearly half were concussions (N = 12). Over 1,500 athletes were observed per year and we conducted 882 ImPACT Pediatric baseline tests with 13 follow-ups. The leading sports for RIO-reportable injury rates was football (year 1) and soccer (year 2). None of the injuries reported required surgery. Concussions were the leading injury in both years of the study, with Year 2 showing only concussions reported. While much is being learned about concussions in professional athletes and those athletes in high school and college settings, a great deal more needs to be understood for the youngest players, especially those who are playing in more uncontrolled settings, including recreational leagues. In addition, parents of youth participants should be adequately informed of concussion information, especially those who are non-English speaking and need materials that are culturally appropriate [17].

Our study did show ImPACT Pediatric was useable with this population of athletes and should be considered as a tool to include with other assessments in terms of diagnosing and treating pediatric concussions. However, in many instances this test takes a substantial amount of time (at least 15 minutes or more) to complete with children, especially those under the age of 10. Finally, our study showed that in order to conduct sports injury assessments in young children it is highly recommended that an ATC be available to collect injury and concussion data and perform appropriate treatment as needed for pediatric injuries.

To the best of our knowledge, this is the first time that researchers collected data on younger children in recreational leagues. Moreover, we used tested tools including RIO to collect injuries and ImPACT to monitor and manage concussions. However, there were limitations to our study. First, we collected data at one large highly controlled athletic facility in west-central Florida which limits generalizability. Due to the sports offered, we only collected data on football, baseball, softball, and soccer, omitting other sports that may have produced additional and different types of injuries such as those in basketball, hockey, lacrosse, etc. Our athletes appeared to lack diversity however we were not permitted to collect any demographic information other than age and gender per sport. We also were not able to collect data on family characteristics that might have influenced the results in terms of parental knowledge, attitudes, and behaviors related to sports injuries.

Future studies should include diverse participants from multiple study venues, include parental data and responses, and additional sports to broaden our understanding of sports injuries in young children. Also, studies related to effective interventions need to be conducted. Some exercise-based injury prevention programs have already been shown to be effective in reducing the number of injuries among young athletes [18]. It is important that interventions are age-specific, sports-specific, and continuously evaluated for efficacy and effectiveness [19, 20].

In order for concussions to decrease, the Centers for Disease Control and Prevention have a focus on changing the culture around concussions [21]. For this to occur, athletes, parents, coaches, and health care professionals all play important roles in decreasing concussions among athletes. Some of these strategies include changing the “win at-all costs” mentality, talking to athletes about concussion, modeling, expecting, and reinforcing safe play, and having concussion information available on every sideline [21].

Other recommendations for the prevention of concussions include ensuring children follow coaching rules for safety, correct protective equipment is worn at all times, and if a concussion is suspected make sure the athlete is removed from play and not return until cleared by a medical professional [22]. Children do require longer recovery times and more conservative treatment approaches [22]. Since soccer has been shown to be an important sport for concussions in our sample, areas for more additional research were assessed [23]. The areas include the benefits and detriments to using headgear, restrictions of heading based on U.S. Soccer’s current policy, benefits of strength training for the neck, properties of the ball and playing surface, and continued studies on the role for education of parents, coaches and healthcare providers for sports related concussions for changing knowledge and behavior.

## Conclusions

The results of our research showed that children, ages 5–11, playing sports in recreational leagues do get injured and that concussions is a leading cause of injury across the sports studied. The RIO and the ImPACT Pediatric tools proved successful in terms of data collection and analysis over the two years of the study.
